# Chitosan extraction from *Amanita phalloides*: yield, crystallinity, degree of deacetylation, azo dye removal and antibacterial properties

**DOI:** 10.3389/fchem.2024.1353524

**Published:** 2024-06-19

**Authors:** Hadia Hemmami, Ilham Ben Amor, Soumeia Zeghoud, Asma Ben Amor, Salah Eddine Laouini, Ali Alsalme, David Cornu, Mikhael Bechelany, Ahmed Barhoum

**Affiliations:** ^1^ Department of Process Engineering and Petrochemical, Faculty of Technology, University of El Oued, El Oued, Algeria; ^2^ Renewable Energy Development Unit in Arid Zones (UDERZA), University of El Oued, El Oued, Algeria; ^3^ Laboratory of Biotechnology Biomaterials and Condensed Materials, Faculty of Technology, University of El Oued, El Oued, Algeria; ^4^ Department of Chemistry, College of Science, King Saud University, Riyadh, Saudi Arabia; ^5^ Institut Européen des Membranes (IEM), UMR 5635, University Montpellier, ENSCM, CNRS, Montpellier, France; ^6^ Gulf University for Science and Technology, GUST, Mubarak Al-Abdullah, Kuwait; ^7^ NanoStruc Research Group, Chemistry Department, Faculty of Science, Helwan University, Cairo, Egypt

**Keywords:** *Amanita phalloids*, chitosan extraction, degree of deacetylation, methylene blue dye removal, response surface model, adsorption optimization, antibacterial activity

## Abstract

Chitosan, a biopolymer obtained from chitin, is known for its remarkable adsorption abilities for dyes, drugs, and fats, and its diverse array of antibacterial characteristics. This study explores the extraction and characterization of chitosan from the mycelium of *Amanita phalloides*. The moisture content, ash content, water binding capacity, fat binding capacity, and degree of deacetylation of the extracted chitosan were determined. The chitosan exhibited a high yield of 70%, crystallinity of 49.07%, a degree of deacetylation of 86%, and potent antimicrobial properties against both Gram-negative and Gram-positive bacteria. The study also examined the adsorption capabilities of chitosan to remove methylene blue (MB) dye by analysing specific factors like pH, reaction time, and MB concentration using the response surface model. The highest degree of MB dye removal was 91.6% at a pH of 6, a reaction time of around 60 min and an initial dye concentration of 16 ppm. This experimental design can be applied for chitosan adsorption of other organic compounds such as dyes, proteins, drugs, and fats.

## Graphical Abstract

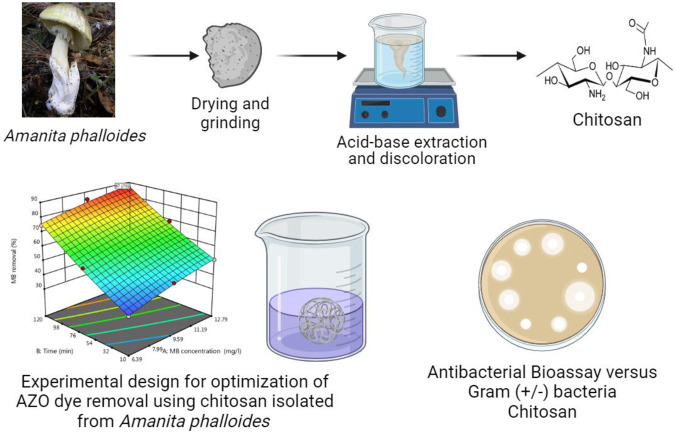

## 1 Introduction

Chitosan, comprised of N-acetyl-D-glucosamine and D-linked D-glucosamine units arranged in a linear polysaccharide structure, boasts several advantageous traits. These characteristics encompass its impressive adsorption properties, increased bioavailability, biodegradability, and lack of toxicity (Torres et al., 2019; Thambiliyagodage et al., 2023). As a derivative of chitin, chitosan emerges through the process of deacetylation, presenting with varying levels of deacetylation. Chitosan is derived from the deacetylation process of chitin found in a variety of organisms such as fungi, crabs, shrimp, squid pens, crawfish, and insects and it demonstrates limited solubility in acidic solutions (Ibrahim and Eid, 2017; Amor et al., 2023). The hydroxyl (-OH) groups present at the C-3 and C-6 positions of chitosan play a key role in its capability to engage with other molecules, improve its solubility in water, and facilitate chemical alterations. The crystallinity, molecular weight, and degree of deacetylation of chitosan are notably affected by the ratio of the two monomer units. This interplay significantly influences chitosan’s antibacterial activity. Chitosan’s versatility, marked by its attributes of biodegradability, biocompatibility, non-toxicity, and absorbency, facilitates its broad utilization across various industries. Its applications span medicine, water treatment, food packaging, wound care, drug delivery systems, textiles, agriculture, and beauty care products (Ibrahim and Eid, 2016; Reshad et al., 2021; Fatullayeva et al., 2022; Thambiliyagodage et al., 2023).

Chitosan exhibits exceptional antimicrobial effectiveness against a wide spectrum of algae, yeasts, bacteria, and fungi. This is attributed to the interaction between the positively charged amino groups of chitosan and the negatively charged molecules present on the surface of bacterial cell membranes (Yilmaz Atay, 2019; Yan et al., 2021). Subsequently, chitosan interferes with DNA replication, leading to cell demise. The chemical modifications of chitosan, such as salinization, ammonium sulfonation, carboxylation, phosphorylation, and quaternization, hold significant potential to alter its antibacterial properties (Ke et al., 2021). Moreover, chitosan’s application extends to dye removal from aqueous solutions, effectively eliminating various cationic and anionic dyes (Li et al., 2021; Nayl et al., 2022). Chitosan and its derivatives boast high absorption capabilities for heavy metal ions and organic dyes, thanks to the abundant presence of -OH and -NH_2_ groups within their polymeric structure. For cationic dyes, chitosan’s negatively charged nature allows it to attract and bond with these dyes through electrostatic interactions. Likewise, with anionic dyes, chitosan’s positive charge enables effective adsorption by attracting and binding with the negatively charged anionic dyes. This adsorption capacity is valuable in various applications such as water treatment, purification processes, and the removal of pollutants from different industrial and environmental settings (Ibrahim and Eid, 2018; Alnemari et al., 2023; Goci et al., 2023).

This study involved the extraction of chitosan from *Amanita phalloides*, followed by several physical characterization techniques to assess its properties, including the extraction yield and degree of deacetylation (DD). Various analysis methods (FTIR, UV-vis, SEM, and XRD) were employed to characterize the chitosan obtained. Various physical characterization techniques including extraction yield, deacetylation degree (DD), moisture content (MC), ash content (AC) measurement, fat binding capacity (FBC) and water binding capacity (WBC) were performed for the synthesized chitosan. The antibacterial efficacy of the chitosan was evaluated against both Gram-positive (e.g., *Staphylococcus aureus*, *Bacillus subtilis*, *Listeria innocuous*) and Gram-negative bacteria (e.g., *Salmonella Typhimurium*, *Pseudomonas aeruginosa*). Notably, the study focused on optimizing treatment conditions using Response Surface Methodology (RSM) to efficiently remove MB dye from wastewater. This optimization process involved adjusting variables like pH, MB concentration, and time to achieve the most effective MB removal. The innovative aspect of this research lies in its use of RSM, a powerful tool for systematic exploration of different parameters and their interactions, thereby enhancing the process optimization. Moreover, the utilization of chitosan derived from mushrooms as an absorbent for removing MB dye not only demonstrates an eco-friendly approach but also introduces a sustainable and renewable source of chitosan.

## 2 Materials and methods

### 2.1 Materials

Hydrochloric acid (HCl, 99%), sodium hydroxide (NaOH, 97%), hydrogen peroxide (H_2_O_2_, 98%), acetic acid (CH₃COOH, 99.5%), and methylene blue (C_16_H_18_ClN_3_S, 82%) dye was purchased from Biochem Chemophara. Bioscan Industrie Algeria gave the Mueller-Hinton agar. *Amanita phalloides* sourced from the local market of El Oued, Algeria (6°52′03″E, 33°22′06″N), was utilized for the extraction of chitin and chitosan. Bacterial samples were obtained from the Algerian Culture Center of Microorganisms.

### 2.2 Extraction of chitin and chitosan from *Amanita phalloides*

**FIGURE 1:**
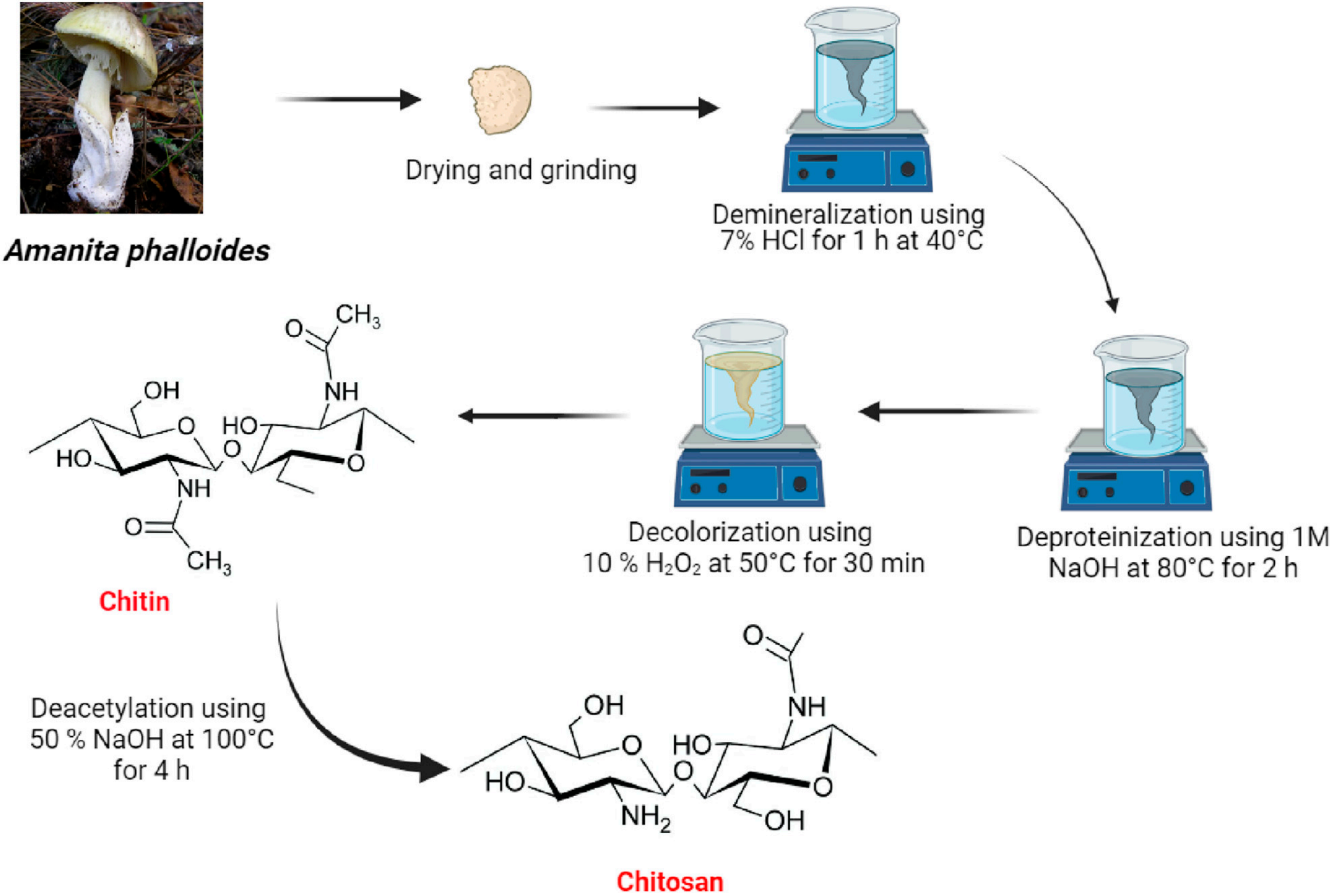
Diagram illustrating the methods involved in extracting chitin and chitosan from *Amanita phalloides.*

Chitin and subsequently chitosan were produced from 40 g of powdered *A. phalloides* through a series of treatments (see Figure 1). Initially, the sample underwent an hour-long treatment with a 7% HCl solution at 40°C to eliminate metals. Subsequently, a 1 M NaOH solution was applied at 80°C for 2 hours, using a solution ratio of 1:10 (w/v), to remove proteins as a step in the deproteinization process. Following a decolorization process using H_2_O_2_ at 50°C for 30 min, the resulting precipitate led to the production of chitin. This chitin was further processed to create chitosan through a deacetylation method, exposing it to a 50% NaOH solution for 4 h at 100°C. The final chitosan was obtained by washing the precipitate repeatedly with distilled water until a neutral pH was achieved.

### 2.3 Physicochemical characterization

FTIR spectra were obtained using a total reflection (ATR) spectrometer (Thermo Scientific-Nicolet iS5) within the range of 400–4,000 cm^−1^. To determine the degree of deacetylation (DD) in chitin and chitosan, FTIR spectroscopy was applied utilizing the formula Eq. 1:

$$DD\left(\%\right)=100-\left[100*\left(A_{1655}/A_{3450}\right)/1.33\right]$$ (1)

Where: A_1655_ and A_3450_ cm^−1^ represent the heights of absorption bands for the amide and hydroxyl groups at 1,655 and 3,450 cm^−1^, respectively. X-ray diffraction (XRD, Rigaku Miniflex 600) was used to examine the crystalline structure of chitosan, employing CuK radiation (40 kV and 30 mA). The crystalline index (CrI) values were calculated using the equation Eq. 2 (Teli and Sheikh, 2012; Ibitoye et al., 2018; Olafadehan et al., 2021b):

$$CrI=\left[I_{110}-I_{am}\right]/I_{110}$$ (2)

Where: I_110_ is the intensity of the crystalline peak at around 2 θ ≅ 20° (002 reflection), and I_am_ is the intensity of amorphous diffraction at 2 θ ≅ 10°–15° (Zhu et al., 2020).

The morphology of chitosan and chitin was examined using a scanning electron microscope (FESEM, Leo Supra 55-Zeiss Inc., Germany). Ultraviolet spectroscopy (UV-2450, Shimadzu, Germany) was employed to record dye absorption spectra in the water treatment experiments.

The chitosan’s ash content was determined through the combustion of the sample in an air atmosphere at 550°C for 3 h. The percentage ash content in a chitosan sample was calculated using Eq. 3:

$$\text{Ash }\%=\left(W2\;/\;W1\right)\;x\;100$$ (3)

where W1 represents the original weight of the chitosan sample, and W2 represents the weight of the residue in grams. The moisture content of the chitosan sample was assessed by vacuum drying it at 110°C for 24 h. The Eq. 4 for determining moisture content (MC, %) is expressed as:

$$\text{MC}\%=\left(W1\;-\;W2\;/\;W1\right)\times 100$$ (4)

where W1 is the weight of the wet sample, and W2 is the weight of the oven-dried sample (Marei et al., 2016).

To measure fat binding capacity (FBC) and water binding capacity (WBC), a centrifuge tube containing 0.5 g of chitosan was initially weighed. For WBC, 10 mL of water was added, while for FBC, soybean oil was used. After combining these substances with chitosan in the centrifuge tube and vortex mixing for 1 min, the mixture stood at 25°C for 30 min. Stirring occurred every 10 min for 5 s, followed by centrifugation at 3,200 rpm for 25 min. After separating the liquid fraction from the solid sediment, the tube was reweighed. WBC and FBC were calculated using the Eqs 5, 6 (Nouri et al., 2016):

$$\text{WBC}\%=\left(\text{weight of water}-\text{bound chitosan}/\text{initial weight of chitosan}\right)\times 100$$ (5)

$$\text{FBC }\%=\left(\text{weight of lipid}-\text{bound chitosan}/\text{initial weight of chitosan}\right)\times 100$$ (6)

### 2.4 Antibacterial bioassay of chitosan

The antibacterial effectiveness of chitosan against various bacterial species, including *Pseudomonas Aeruginosa* (ATCC9027), *Bacillus Subtiliis* (ATCC6633), *Listeria Innocua* (CLIP74915), *Staphylococcus Aureus* (ATCC6538), and *Salmonella Typhimurium* (ATCC14028), was assessed using the agar well diffusion technique. Wells of 6 mm in diameter were created on agar plates using a sterile stainless steel cork borer. The culture plates were then prepared and inoculated with 100 μL of a 24-hour-old bacterial broth culture. Chitosan’s bactericidal activity was examined at various concentrations (1%, 4%, and 8%) in acetic acid, with ciprofloxacin (CIP-5) used as the reference standard for comparison. The plates were calibrated using a 5 μL solution of chitosan. Following a 24-h incubation period at 37°C, inhibitory zones were observed.

### 2.5 Adsorption studies on chitosan

The experiment was designed using the Box Behnken design and the statistical program Design Expert ver.15 by Response Surface Methodology. Table 1 outlines the factors considered in this research. Chitosan was added to 0.5 g flasks containing 30 mL of dye solution at varying concentrations (ranging from 9.59 to 15.99 ppm) and across predetermined pH values (ranging from 2 to 10) for different durations (from 10 to 60 min) to achieve equilibrium. The mixture was agitated in an orbital shaker at 150 rpm and maintained at a temperature of 25°C. Subsequently, the solid phase was separated by centrifugation at 6,000 rpm. A UV-vis spectrophotometer was utilized to compare the concentration of the MB dye before and after the adsorption procedures. Additionally, 0.1 M NaOH and 0.1 M HCl were employed to adjust the solution’s pH during the experiment. The percentage of dye removal capacity was calculated using the Eq. 7 provided below (Ben Amor et al., 2022).

$$Dye\;removal\left(\%\right)=\frac{\left(C_{0}-C_{e}\right)}{C_{0}}\times 100$$ (7)

**TABLE 1:** Design matrix for various factor values and experimental responses.

Run no.	Experimental factors			Response (MB removal)	
	pH	MB conc. (ppm)	Time (min)	Actual value	Predicted value
1	10	15.99	35	57.1	56.65
2	2	12.79	60	57.38	57.77
3	6	12.79	35	87.52	85.52
4	2	12.79	10	49.34	49.25
5	6	15.99	60	91.23	91.60
6	6	12.79	35	84.52	85.52
7	2	9.59	35	45.36	45.82
8	6	9.59	60	82.65	81.80
9	6	15.99	10	89.14	89.99
10	6	9.59	10	79.92	79.55
11	6	12.79	35	84.52	85.52
12	2	15.99	35	61.28	60.52
13	10	12.79	60	51.81	51.90
14	10	12.79	10	56.95	56.56
15	10	9.59	35	50.36	51.12
Factors and levels for experimental design					
Factors		Codes	−1.0	0.0	1.0
pH		A	2	6	10
MB concentration (ppm)		B	9.59	12.79	15.99
Time (min)		C	10	35	60

Co and Ce in the formula represent the initial (starting) and equilibrium dye concentrations in mg.L^−1^ (ppm), respectively.

The quadratic Eq. 8 can be stated as follows in the Box-Behnken Design, with 03 factors (Ben Amor et al., 2023):

$$Y=\beta_{0}+\sum_{j=1}^{m}\beta_{j}x_{j}+\sum_{j=1}^{m}\beta_{jj}{x_{j}}^{2}+\sum \sum_{i<j=2}^{m}\beta_{ij}x_{i}x_{j}+\varepsilon$$ (8)

Where ε represents the error, $x_{i}$ and $x_{j}$ represent variables. The linear, binominal, and combination effects are represented by the coefficients $\beta_{\dot{i}}$ , $\beta_{\text{ii}}$ , and $\beta_{ij}$ , respectively. $\beta_{0}$ is a constant coefficient, and m denotes the total of the factors under study (Mondal and Purkait, 2018).

## 3 Results and discussion

### 3.1 Characteristics of chitosan

The production of chitosan extracted from *A. phalloides* is a critical metric that indicates the efficiency of the extraction process. In several studies it has been reported that the chitosan content extracted from different insects ranges from 2% to 79% (Mohan et al., 2020; Zainol Abidin et al., 2020). In the course of this study, the extraction yield of chitin and chitosan from *A. phalloides* was determined to be 52.5% and 70%, respectively. The yield of chitosan from *A. phalloides* indicates a relatively efficient and productive extraction process. Achieving a higher yield in the extraction process is generally favourable because it ensures a greater amount of chitosan, which can be used in different applications across different industries.

Amanita phalloides chitosan exhibits notable Water Binding Capacity (WBC) at approximately 671% and a considerable Fat Binding Capacity (FBC) of around 253%. In comparison, commercial chitosan shows WBC capabilities similar to extracted chitosan but tends to have lower FBC. Many commercially available chitosan display WBC and FBC capacities ranging between 458%–805% and 314%–535%, respectively (Kumari et al., 2017). The term “ash contents” refers to the inorganic residue resulting from the complete decomposition of chitosan when subjected to heating in the presence of air. The concentration of chitosan ash is a crucial factor in assessing the efficacy of calcium carbonate removal during the demineralization phase. High-quality chitosan typically maintains an ash concentration of less than 1% (Ibitoye et al., 2018). In Table 2, the chitosan isolated from *A. phalloides* is reported to have an ash concentration of 6.7%, indicating that chitosan from *A. phalloides* possesses superior quality. Additionally, chitosan derived from *A. phalloides* is found to have a moisture content of 13.9%.

**TABLE 2:** Characteristics of the chitosan extract.

Characteristics	Value (%)
Yield (Y)	70
Ash contents (AC)	6.7
Moisture content (MC)	13.9
Fat binding capacity (FBC)	253
Water binding capacity (WBC)	671
Crystallinity Index (CrI)	49.07

### 3.2 Crystallinity of chitin and chitosan

**FIGURE 2:**
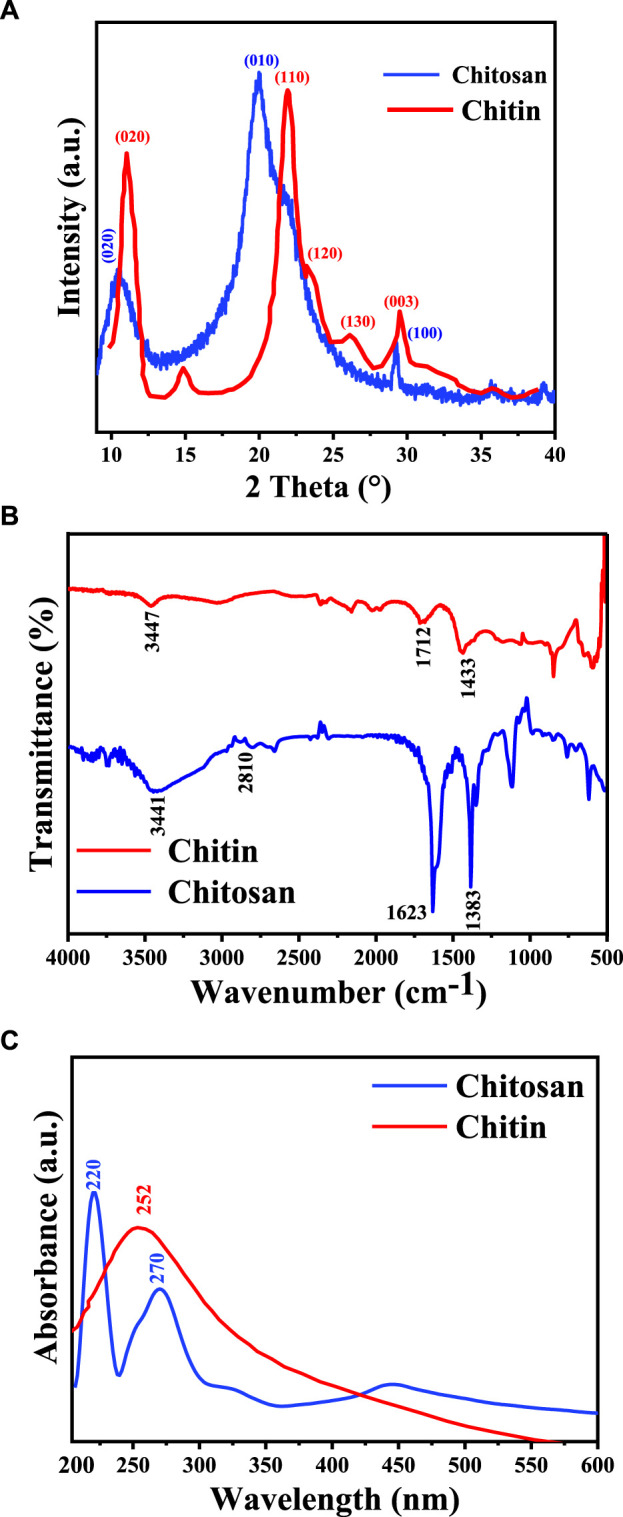
Chemical analysis of chitosan and chitin extracted from *Amanita phalloides*: **(A)** XRD diffraction; **(B)** FTIR spectra; and **(C)** UV-vis spectra.

XRD spectra of chitin and chitosan, depicted in Figure 2A, unveil intricate patterns indicative of their crystalline and semi-crystalline nature. Chitin’s XRD pattern showcases distinctive boundary peaks at 11.04°, 14.89°, 21.94°, 23.16°, 26.18°, and 29.26°, consistent with prior studies (Kaya et al., 2015a; Raut et al., 2016; Olafadehan et al., 2021b). Conversely, XRD pattern of chitosan shows three peaks at 10.57°, 20.30°, and 29.26°, with a notably lower intensity observed at 10.57°. This reduction in peak intensity is attributed to the formation of intramolecular hydrogen bonds post-deacetylation, a phenomenon well-documented in the literature (Kaya et al., 2015b). Crystalline reflections characteristic of chitin and semi-crystalline features indicative of chitosan are discerned at 2θ = 20.30° and 2θ = 10.57°, respectively. CrI values were calculated using Eq. 2, as described by Olafadehan et al. (2021b), Ibitoye et al. (2018), and Teli and Sheikh (2012). In this study, CrI values for chitin and chitosan stand at 70.01% and 49.07%, respectively. These findings align with previous research, which reported CrI values for chitin ranging between 50% and 90% and for chitosan between 20% and 70%, underscoring the inherent variability in crystallinity within these polymers (Ibitoye et al., 2018; Mohan et al., 2020).

In a study by Kaya et al. (2015c), CrI values for wasp-extracted chitin were reported to be 50% for Vespula germanica, 69.88% for Vespa crabro, and 53.92% for Vespa orientalis, highlighting variability in crystallinity among different chitin sources. Similarly, Hajji et al. (2014) found that chitin prepared from shrimp and crab shells exhibited crystallinity rates of 64.1% and 67.8%, respectively, while chitosan derived from the same sources displayed crystallization rates of 31.9% and 35.2%. These findings indicate the influence of source material on the crystalline properties of chitin and chitosan. Structural modifications introduced during the deacetylation process contribute to chitosan’s lower CrI, characterized by disrupted polymer chain organization, increased hydrophilicity, and enhanced flexibility, factors that influence the formation of crystalline domains. Furthermore, it's important to note that processing parameters such as temperature, pH, and solvent choice play crucial roles in determining the final crystalline structure of chitosan, adding additional layers of complexity to its crystallinity behaviour and emphasizing the need for meticulous control in manufacturing processes.

### 3.3 Degree of deacetylation of chitin and chitosan

Utilizing FTIR spectroscopy, the degree of deacetylation in chitosan was assessed (Figure 2B). Notably, the peaks at 3,460 cm^−1^ in both chitin and chitosan spectra are attributed to the stretching vibrations and hydrogen bonding among NH_2_ and -OH groups (Amor et al., 2023). Specific characteristics observed in the chitin spectrum include the band at 1,712 cm^−1^, indicating the expansion of the C=O hydrogen group bonded to N-H, and the band at 1,433 cm^−1^, suggesting a distinct hydrogen bond between the C=O and the hydroxyl-methyl group of the chitin residue [19]. Conversely, the chitosan spectrum displays distinctive peaks, such as the band at 2,810 cm^−1^ (stretching of H-C polysaccharide bonds), 1,623 cm^−1^ (amide I indicating the removal of the acetyl group), and 1,338 cm^−1^ (amide II representing bending -NH_2_) (Qiao et al., 2021). The degree of deacetylation (DD) calculated via FTIR analysis was 16% for chitin and 86% for chitosan. It's important to note that the value of deacetylation can varying sample preparation methods, and chitosan resources.

### 3.4 UV-Vis spectra of chitin and chitosan

In Figure 2C, the UV-Vis spectra of chitin and chitosan obtained from *A. phalloides* are presented. Notably, each spectrum exhibits unique absorption bands: specifically, chitosan displays a prominent absorption band at 252 nm, while chitin exhibits absorption bands at 220 nm and 270 nm. These absorption peaks correspond to π-π* transitions of electrons within the ring system and n-π* transitions of electrons in the oxygen or nitrogen atoms attached to the ring system, respectively, following the removal of the acetyl group. These findings align with prior research in the field (Fatullayeva et al., 2022; Tarique et al., 2023). The difference in the UV-Vis absorption patterns between chitin and chitosan, particularly in the observed wavelengths, highlights the alteration in the molecular structure due to the deacetylation process and changing of the chemical structure from chitin to chitosan.

### 3.5 Morphological analysis of chitin and chitosan

**FIGURE 3:**
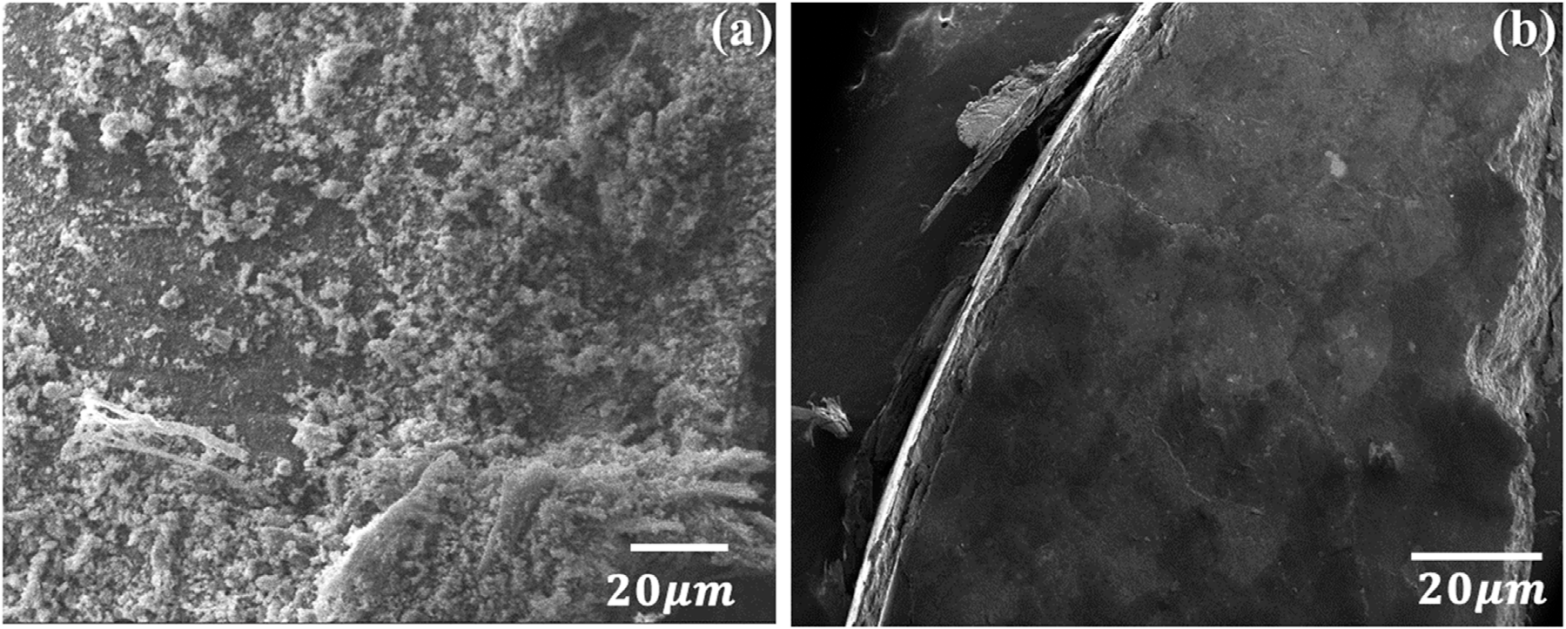
SEM images of **(A)** chitin and **(B)** chitosan extracted from *Amanita phalloides*.

The morphological analysis of chitin (Figure 3A) and chitosan (Figure 3B), observed through a scanning electron microscope (SEM) reveals distinct differences in their structures. Chitin demonstrates an irregular structure, characterized by a rough surface, whereas chitosan showcases a transformed surface, displaying a smoother polymer structure. This observation aligns with findings from multiple earlier studies (Olafadehan et al., 2021a; Son et al., 2021; Piekarska et al., 2023). In contrast, chitosan, derived from chitin through deacetylation, presents a modified structure with a smoother surface, indicative of its altered polymer nature. This difference in morphology between chitin and chitosan serves as a visual confirmation of the structural changes brought about by the deacetylation process, which are consistent with previous research findings.

### 3.6 Antibacterial activities of chitosan

**FIGURE 4:**
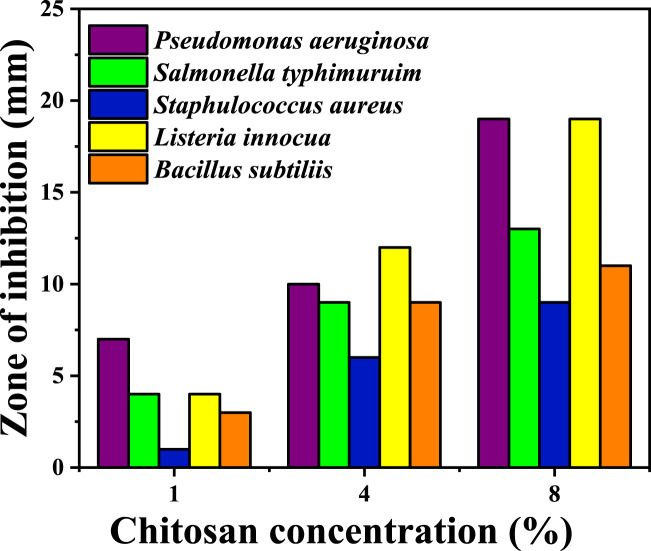
Antibacterial activity of chitosan at various concentrations against different bacteria. The values of the chitosan solutions were subtracted from the inhibition zone of acetic acid. (Every test was carried out in triplicate. The values were expressed as means ± standard deviation (SD) (*n* = 3).

Antibacterial activity of chitosan against both Gram-positive (Gram+) and Gram-negative (Gram -) bacteria is illustrated in Figure 4; Table 3, revealing an increase in the zone of inhibition as the concentration of chitosan rises. For Gram-negative bacteria, such as *P. aeruginosa* and *Salmonella typhimurium*, the zone of inhibition increased with higher chitosan concentrations. For instance, at a chitosan concentration of 1 wt%, the zones of inhibition were 7 ± 0.1 mm and 4 ± 0.3 mm against *P. aeruginosa* and *S. typhimurium*, respectively. However, at 8 wt% concentration, these values increased to 19 ± 0.3 mm and 13 ± 0.25 mm, indicating a notable increase in the inhibitory effect against these Gram-negative bacteria. Similarly, for Gram-positive bacteria, higher chitosan concentrations resulted in more substantial zones of inhibition. *Pseudomonas aeruginosa* and *Listeria innocua* demonstrated higher sensitivity to chitosan, especially at higher concentrations. At 8 wt% chitosan concentration, the zone of inhibition against *P. aeruginosa* and *L. innocua* was measured at 19 ± 0.3 mm and 19 ± 0.17 mm, respectively.

**TABLE 3:** Zone of inhibition of the extracted chitosan.

Sample	Conc	Zone of inhibition^a^ (mm)				
		Gram-negative bacteria		Gram-positive bacteria		
		*Pseudomonas aeruginosa*	*Salmonella typhimuruim*	*Staphulococcus aureus*	*Listeria innocua*	*Bacillus subtiliis*
Chitosan (is dissolved in acetic acid)^b^	1 wt%	7 ± 0.1	4 ± 0.3	1 ± 0.1	4 ± 0.1	3 ± 0.2
	4 wt%	10 ± 0.1	9 ± 0.1	6 ± 0.25	12 ± 0.1	9 ± 0.1
	8 wt%	19 ± 0.3	13 ± 0.25	9 ± 0.15	19 ± 0.17	11 ± 0.25
Ciprofloxacin (CIP-5)	50 µg	24 ± 0.4	16 ± 0.15	16.5 ± 0.2	24 ± 0.3	24 ± 0.3

^a^ The value of three measurements was used to calculate the zone of inhibition.

^b^ The values of the chitosan solutions were subtracted from the inhibition zone of acetic acid.

Chitosan primarily exerts its antibacterial effect through electrostatic interactions with the surface of bacterial cells, inducing increased permeability in the bacterial membrane (Alhamad et al., 2023). This interaction occurs between the positively charged protonated amino group of chitosan and the negatively charged molecules on the bacterial cell membrane (Ardean et al., 2021). Research by Zeinab Abedian and colleagues (Abedian et al., 2020) assessed the antibacterial activity of commercial chitosan with a high degree of deacetylation (94%) and low molecular weight against various spoilage pathogens. The study demonstrated notable variations in sensitivity between Gram-positive and Gram-negative bacteria when exposed to chitosan. Multiple factors are crucial in determining the antibacterial activity of chitosan, such as the bacterial strain, growth stage, molecular weight, concentration, environmental temperature, and pH level (Ardean et al., 2021). Chitosan exhibits an affinity for the surfaces of negatively charged cells, like Gram-negative bacteria, when present in low quantities, reducing cell membrane permeability, resulting in cell death. The positively charged amino groups of chitosan create a protective coating on the cell surface, impeding the entry of intracellular components (Ibitoye et al., 2018). In contrast, for Gram-positive bacteria, the electrostatic repulsion between the positively charged chitosan and bacterial cells impedes agglutination (Abedian et al., 2020; Ke et al., 2021). In a study investigation into the fractional weight and concentration of chitosan against *E. coli* demonstrated that high concentrations (>200 ppm) exhibited effective antibacterial properties, while low concentrations (20 ppm) displayed a weaker effect (Ardean et al., 2021). At higher concentrations, the bactericidal effects may be due to bacterial flocculation, whereas low concentrations may Favor bacterial proliferation, promoting survival (Antaby et al., 2021). This difference in effect might be linked to the antibacterial mechanisms associated with varying chitosan concentrations.

### 3.7 Experimental design for dye removal

Table 1 displays the experimental outcomes related to the removal of MB dye and provides a comprehensive comparison between anticipated and actual results. Each row in this table outlines a specific experimental setup, detailing the values assigned to key parameters such as pH, MB concentration (ppm), and time (min). Moreover, the table encompasses both the anticipated and observed MB removal percentages under these set conditions. Notably, the comparison between the predicted and actual values demonstrates a remarkable consistency, affirming the accuracy of the proposed model. Experimental setups 5 and 9 exhibit notably high MB removal percentages, aligning closely with the predicted values of 91.60% and 89.99%, respectively. Conversely, setups 2, 4, and 13 demonstrate lower MB removal percentages, corresponding to the expected values of 57.38%, 49.34%, and 51.81%, respectively. This congruence between the predicted and observed responses emphasizes the reliability of the model in estimating MB removal efficiency across diverse experimental conditions.

The analysis of variance (ANOVA) conducted for MB dye removal using chitosan has resulted in a highly accurate quadratic polynomial model equation. This equation effectively correlates the responses with the variables being investigated (as detailed in Table 4). The exceptionally high coefficient of determination (*R* ^2^ = 0.9977) indicates an almost perfect alignment between the predicted and actual values, while the adjusted *R* ^2^ value (0.9934) further underscores the model’s exceptional accuracy. The significant F-value (236.59, *p* < 0.0001) highlights the overall reliability and importance of the model. In the ANOVA, each factor (pH, MB concentration, and time) and their interactions were thoroughly examined. Factors A (pH), B (MB concentration), AB (interaction between pH and MB concentration), AC (interaction between pH and time), and A^2^ (pH square term) exhibited substantial impact with *p*-values <0.05. However, factors C (time) and BC (interaction between MB concentration and time) displayed slightly higher *p*-values, though still within a reasonably acceptable range (0.1059 and 0.8265, respectively). The coefficients within the quadratic polynomial model equation further elucidate the magnitude and direction of influence each factor holds over the MB removal response.

**TABLE 4:** Adsorption using ANOVA variance analysis.

Source	Sum of squares	Degree of freedom	Mean square	F -value	*p*-value
Model	4,087.90	9	454.21	236.59	< 0.0001
A	1.02	1	1.02	0.5326	0.4983
B	204.63	1	204.63	106.58	0.0001
C	7.45	1	7.45	3.88	0.1059
AB	21.07	1	21.07	10.97	0.0212
AC	43.43	1	43.43	22.62	0.0051
BC	0.1024	1	0.1024	0.0533	0.8265
A^2^	3,764.40	1	3,764.40	1960.77	< 0.0001
B^2^	0.0156	1	0.0156	0.0081	0.9317
C^2^	0.2895	1	0.2895	0.1508	0.7138
Residual	9.60	5	1.92		
Lack of fit	3.60	3	1.20	0.3999	0.7704
Pure error	6.00	2	3.00		
Total	4,097.50	14			
*R* ^2^	0.9977				
*R* ^2^ Adjusted	0.9934				

The analysis has shown that a quadratic polynomial model’s equation, which expresses the responses in coded factors, links the responses with the factors under study. This model is given below in Eq. 9. The terms “sign” (positive or negative) and “coefficient” (representing the kind and strength of the influence on the response) are used in the equation to accurately characterize the effect of each element.

$$\text{MB removal }\left(\%\right)=85.52+0.3575\;A+5.06\;B+0.9650\;C-31.93\;A^{2}\;-0.0650\;B^{2}+0.2800\;C^{2}-2.29\text{ AB}-3.29\text{ AC}-0.1600\text{ BC}$$ (9)

A is pH, B is MB concentration (ppm), and C is the Time (min).

**FIGURE 5:**
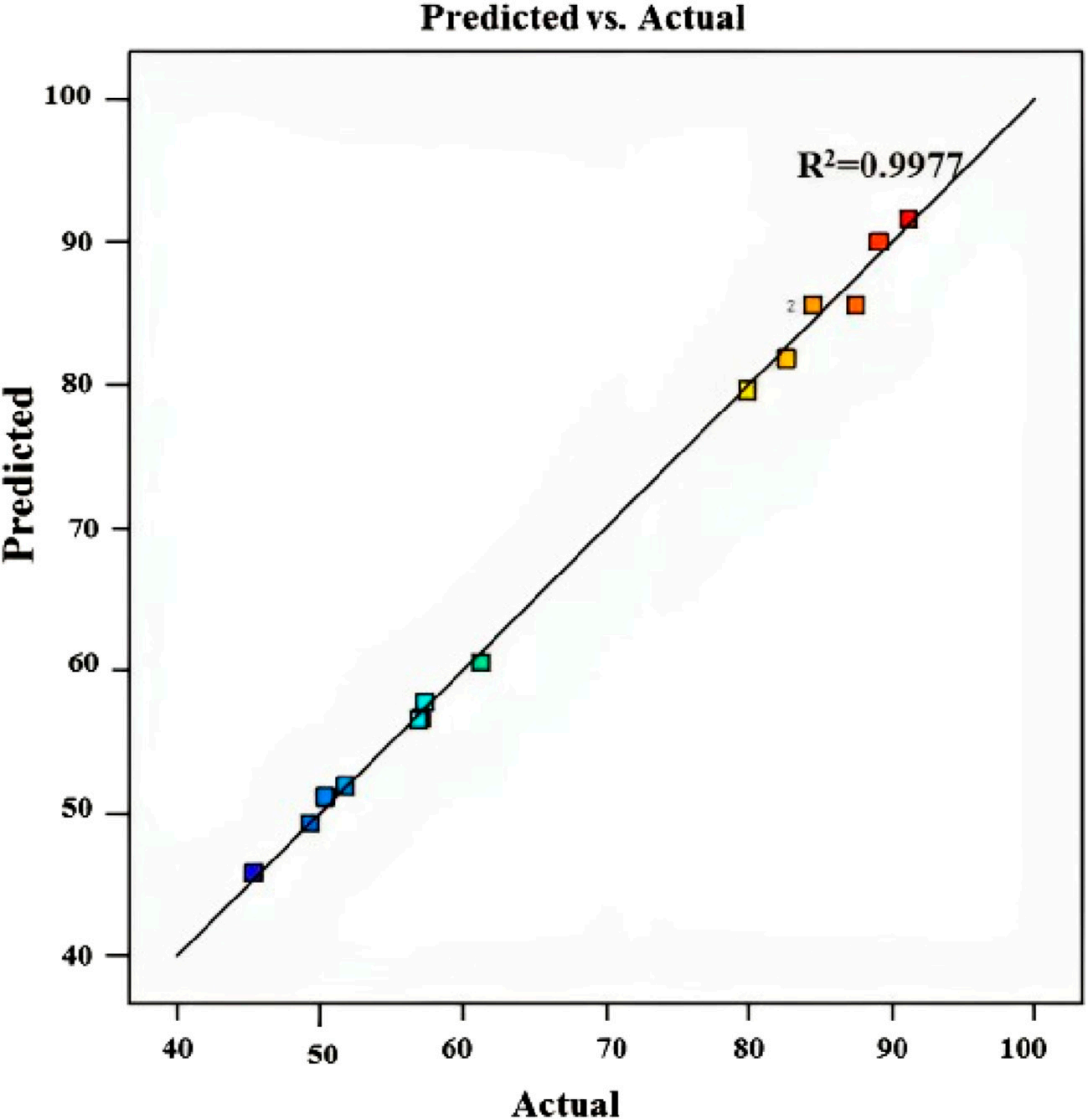
Predicted vs. experimental values for MB dye removal using chitosan extracted from *Amanita phalloides*.

Figure 5 displays the expected versus actual values for the MB dye removal reaction, yielding an *R* ^2^ value of 0.9977 (Table 4), revealing that the predicted values align closely with the experimental values, affirming the accuracy of the model’s predictions. This suggests that the model developed is effective in estimating the efficiency of chitosan in removing MB dye under various conditions, providing insights into optimizing the removal process for enhanced effectiveness.

**FIGURE 6:**
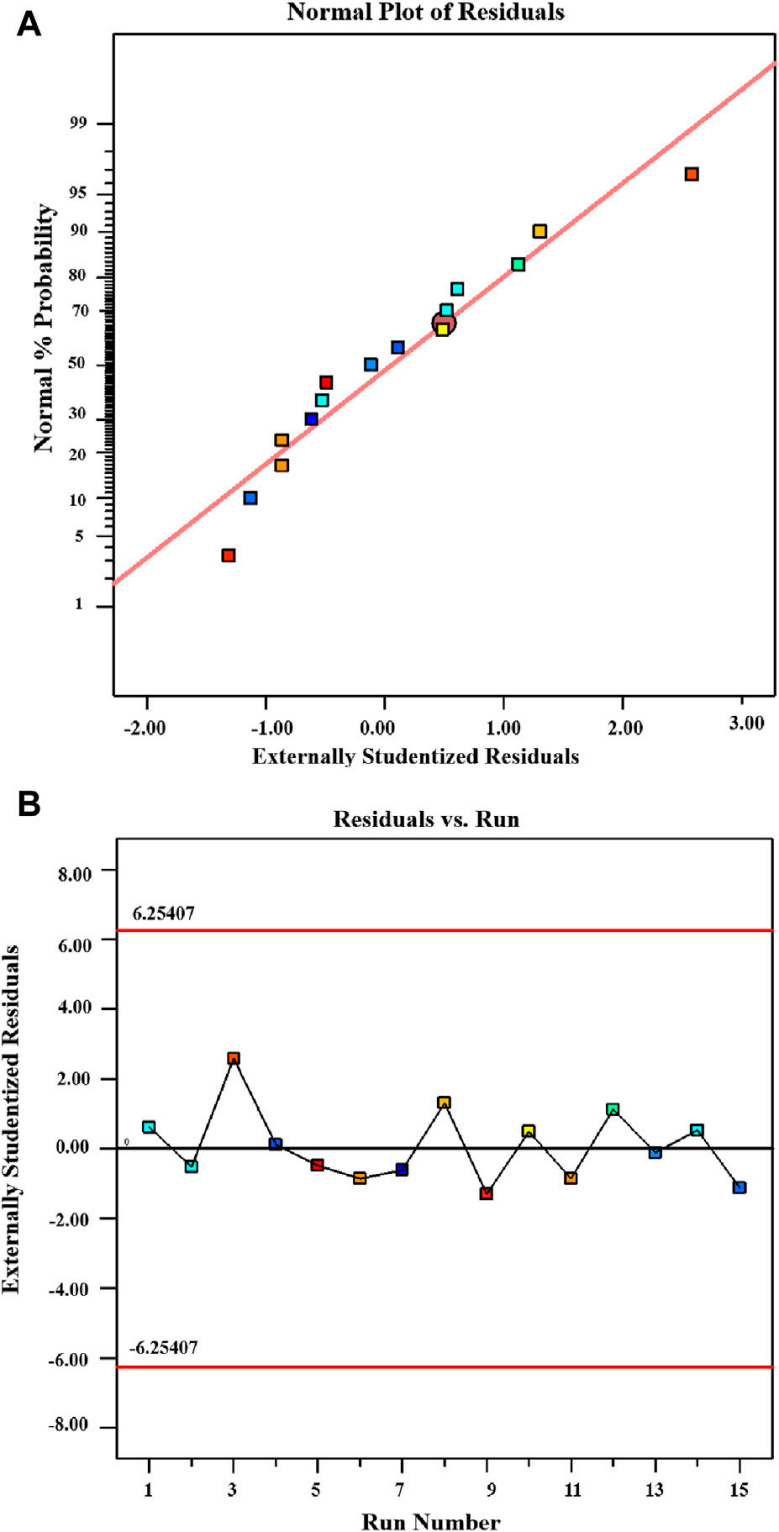
Experimental design for dye removal **(A)** Polt displaying the externally studentized residual along with normal probability. **(B)** Polt depicting the predicted value against the externally studentized residuals for MB dye removal utilizing chitosan.

Figure 6A presents a normal probability plot that is particularly useful in determining the distribution pattern of the data (Moria et al., 2022). The lack of conspicuous deviations in the plot signifies that the data aligns well with the assumptions of the statistical model. In Figure 6B, the residuals are displayed along with the run numbers in what’s known as an outlier t-plot. The red lines at the top (6.25407) and bottom of the figure (−6.25407) indicate the 95% confidence interval for the control range. Remarkably, all data points fall within this allowed range (from 6.25407 to–6.25407), demonstrating consistency with expectations. Additionally, the histogram of the residual data shows a lack of any discernible structure or pattern, suggesting a random and normal distribution of residuals. The absence of a specific shape or structure in the distribution implies that there is no systematic connection or pattern among the residuals.

**FIGURE 7:**
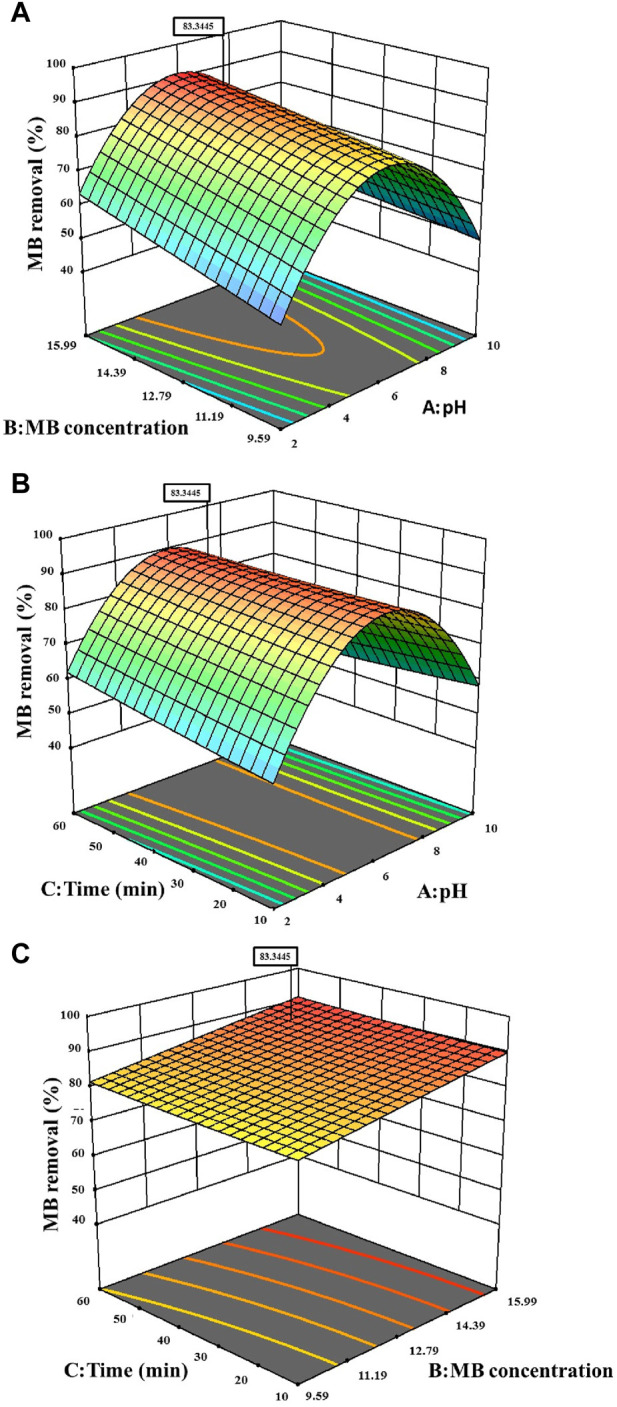
3-D Surface contour plot for the effects on MB dye removal **(A)** MB concentration versus pH, **(B)** Time versus pH **(C)** Time versus MB concentration.

Process parameters (pH, MB concentration, and time) are intentionally varied according to a pre-established plan, often utilizing a response surface design, to assess their influence on the targeted response variable. Figure 7 portrays 3D plots of the response surfaces, illustrating the individual impacts of the three independent parameters (pH, MB concentration, and time) on MB removal. Analysing the response surface aids in comprehending the singular and combined influences of these input factors on the outcomes. The curves derived from the plots demonstrate that the efficiency of MB removal increases with a rise in the initial concentration of MB from 12.79 to 15.99 ppm, and within a pH range of 4.5–6.5. The peak MB removal efficiency reached 83.34% at a pH of 7.67%, an MB concentration of 15.54 ppm, and a contact time of 46.12 min.

**FIGURE 8:**
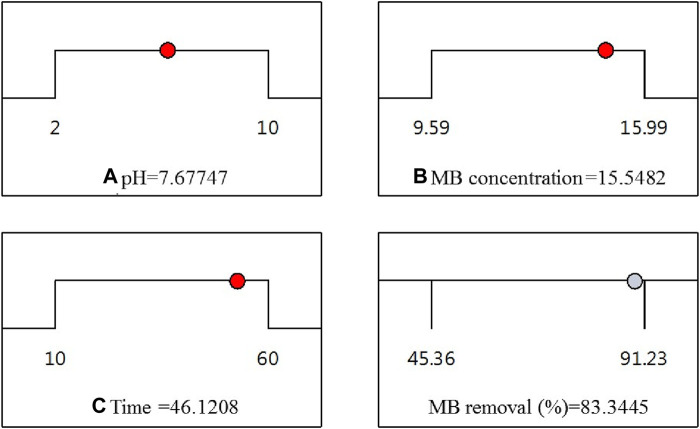
Desirability ramp for optimization.

Throughout this study, all factor ranges were confined between their minimum (1) and maximum (+1) values, aiming to optimize the maximization of MB elimination. The graphical representation in Figure 8 demonstrated a maximum desirability of 1.00 using the preset optimization criteria. With these established conditions, the observed maximum MB Removal (%) of 83.34% corresponded to a pH (A) of approximately 7.67, an MB concentration (B) around 15.54 ppm, and a reaction time (C) close to 46.12 min. Under the optimized Response Surface Methodology conditions, the highest degree of MB dye removal was found to be 91.6%. This occurred at a pH of 6, a MB concentration of approximately 15.99 ppm, and a reaction time of around 60 min.

### 3.8 Evaluation of the previous research

Chitosan is acknowledged for its efficacy as an adsorbent, adept at the removal of diverse pollutants including heavy metals, organic dyes, and microbial contaminants. Its reusability stands out as a key advantage in wastewater treatment, promising cost-effectiveness and sustainability (da Silva Alves et al., 2021). Numerous studies have delved into the reusability of chitosan-based adsorbents, consistently showing that chitosan can be regenerated and reused multiple times without compromising its adsorption capacity or performance (Dago-Serry et al., 2024; Kaczorowska and Bożejewicz, 2024). Typically, the regeneration process involves desorbing the adsorbed contaminants from the chitosan matrix using appropriate desorbing agents, followed by washing and drying for subsequent reuse (Soares et al., 2021). However, handling contaminated chitosan, especially when loaded with basic dyes, requires careful consideration to ensure safe and effective management of the material and its associated contaminants. Here are some key considerations.

(1) Storage: Contaminated chitosan should be stored in designated containers or areas clearly labelled to indicate the presence of hazardous substances. Storage facilities must be secure and inaccessible to unauthorized individuals to prevent accidental exposure (Moder et al., 2007).
(2) Disposal: Disposing of contaminated chitosan should adhere to local regulations and guidelines for handling hazardous waste. This typically involves segregating contaminated materials, proper packaging, and transportation to authorized disposal facilities equipped to handle hazardous waste (Goel, 2017).
(3) Treatment: Depending on the contamination’s nature and extent, treatment options like chemical or thermal treatment, incineration, or biological degradation may be considered to deactivate or remove contaminants from the chitosan matrix before disposal. These methods should be chosen for their effectiveness in decontaminating the material while minimizing environmental impact (González-Martínez et al., 2019).
(4) Recycling: Contaminated chitosan may undergo regeneration or recycling processes to recover valuable components or resources. This often entails desorbing contaminants, followed by purification or regeneration of the chitosan matrix for reuse in subsequent applications (Bhatt et al., 2023).

In Table 5, a comparative analysis of the efficiency in removing MB dye by chitosan derived from various sources is presented, shedding light on the absorption rates achieved across different experimental conditions. The findings suggest that chitosan extracted from *Fenneropenaeus indicus* demonstrated a relatively higher MB removal percentage, reaching 93.2% at a low MB concentration of 10 ppm, with a pH of 7, a reaction volume of 50 mL, and a 24-h reaction time. In contrast, the chitosan extracted from *A. phalloides*, the focus of this study, displayed a high MB removal percentage of 91.6% at a higher MB concentration of 15.99 ppm, a pH of 6, and a shorter reaction time of 1 h, with a reaction volume of 30 mL. However, chitosan obtained from other sources, namely, *Blaps lethifera*, *Pimelia fernandezlopezi*, and *Musca domestica*, exhibited lower MB removal percentages. They achieved 88.7%, 47.1%, and 29.0% removal rates, respectively, at a low MB dye concentration of 12.79 ppm, a pH of 7, and a reaction time of 2 h. Our results showed that the extracted chitosan showed high adsorption capacity for the pollutant present in wastewater. The unique physicochemical properties of chitosan, such as its positively charged functional groups, enable it to effectively adsorb diverse contaminants through various mechanisms, including electrostatic interactions, hydrogen bonding, and surface complexation (da Silva Alves et al., 2021).

**TABLE 5:** Comparing the outcomes of MB dye removal efficiency of chitosan prepared from various sources with other studies.

Source of chitosan	Extraction conditions	MB removal (%)	Ref.
*Fenneropenaeus indicus*	Chitosan mass: 4 g; MB concentration: 10 ppm, pH = 7, time: 24 h	93.2	Arunachalam (2021)
*Blaps lethifera*	Chitosan mass 1 g; MB concentration 12.79 ppm; pH 7, volume 30 mL, time: 120 min	88.7	Ben Amor et al. (2023)
*Pimelia fernandezlopezi*		47.1	
*Musca domestica*		29.0	
*Amanita phalloides*	Chitosan mass 0.5 g, MB concentration 15.99 ppm; volume: 30 mL, pH 6; time 60 min	91.6	This work

## 4 Conclusion

Extraction of chitin and chitosan from *A. phalloides* exhibited a yield of 52.5% and 70%, respectively. Several parameters were investigated, encompassing the degree of deacetylation (DD), fat binding capacity (FBC), water binding capacity (WBC), as well as moisture content and ash content. Notably, the antibacterial activity of chitosan revealed higher efficacy against Gram-positive bacteria compared to Gram-negative strains. Employing Response Surface Methodology based on the Box-Behnken design, the impact of specific conditions (time, pH, methyl blue concentration) on the removal of MB was rigorously examined. The high R value (*R* ^2^ = 0.99) underscores the model’s relevance in MB dye removal. Under the optimized Response Surface Methodology conditions, the highest degree of MB dye removal was found to be 91.6%. This occurred at a pH of 6, a MB concentration of approximately 15.99 ppm, and a reaction time of around 60 min. These findings suggest that the procedure for chitosan extraction from *A. phalloides* is a cost-effective, eco-friendly, and biodegradable approach for wastewater treatment. Response Surface Methodology emerges as a valuable statistical tool for enhancing chitosan’s efficacy in removing diverse organic pollutants. Overall, the study highlights the potential of chitosan sourced from this fungal origin in addressing azo dye removal from wastewater and exploring its antibacterial applications. The study’s successful demonstration of chitosan’s prowess in dye removal and antibacterial properties promises significant environmental and health benefits by offering an eco-conscious solution for wastewater treatment.

## Data Availability

The original contributions presented in the study are included in the article/Supplementary material, further inquiries can be directed to the corresponding author.

## References

[B1] Abedian Z. Jenabian N. Moghadamnia A. Zabihi E. Pourbagher R. Rajabnia M. (2020). Antibacterial activity of high-molecular-weight and low-molecular-weight chitosan upon oral pathogens. Int. J. Infect. Dis. 101, 46–47. 10.1016/j.ijid.2020.09.154 31142988 PMC6519183

[B2] Alhamad A. A. Zeghoud S. Amor I. B. Hemmami H. (2023). Chitosan-based hydrogels for wound healing: correspondence. Int. J. Surg. 109, 1821–1822. 10.1097/js9.0000000000000414 37076129 PMC10389636

[B3] Alnemari A. M. Moustapha M. E. Hassan A. A. Salah D. (2023). Chitosan nano-composites applications for water remediation. Cogent Eng. 10, 2220498. 10.1080/23311916.2023.2220498

[B4] Amor I. B. Hemmami H. Laouini S. E. Abdelaziz A. G. Barhoum A. (2023). Influence of chitosan source and degree of deacetylation on antibacterial activity and adsorption of AZO dye from water. Biomass Convers. Biorefinery, 1–11. 10.1007/s13399-023-03741-9

[B5] Antaby E. Klinkhammer K. Sabantina L. (2021). Electrospinning of chitosan for antibacterial applications—current trends. Appl. Sci. 11, 11937. 10.3390/app112411937

[B6] Ardean C. Davidescu C. M. Nemeş N. S. Negrea A. Ciopec M. Duteanu N. (2021). Factors influencing the antibacterial activity of chitosan and chitosan modified by functionalization. Int. J. Mol. Sci. 22, 7449. 10.3390/ijms22147449 34299068 PMC8303267

[B7] Arunachalam K. (2021). Bio-adsorption of methylene blue dye using chitosan-extracted from Fenneropenaeus indicus shrimp shell waste. J. Aquac. Mar. Biol. 10, 146–150. 10.15406/jamb.2021.10.00316

[B8] Ben Amor I. Hemmami H. Laouini S. E. Mahboub M. S. Barhoum A. (2022). Sol-gel synthesis of ZnO nanoparticles using different chitosan sources: effects on antibacterial activity and photocatalytic degradation of AZO Dye. Catalysts 12, 1611. 10.3390/catal12121611

[B9] Ben Amor I. Hemmami H. Laouini S. Zeghoud S. Benzina M. Achour S. (2023). Use of insect-derived chitosan for the removal of methylene blue dye from wastewater: process optimization using a central composite design. Materials 16, 5049. 10.3390/ma16145049 37512323 PMC10383991

[B10] Bhatt P. Joshi S. Bayram G. M. U. Khati P. Simsek H. (2023). Developments and application of chitosan-based adsorbents for wastewater treatments. Environ. Res. 226, 115530. 10.1016/j.envres.2023.115530 36863653

[B11] Dago-Serry Y. Maroulas K. N. Tolkou A. K. Kokkinos N. K. Kyzas G. Z. (2024). How the chitosan structure can affect the adsorption of pharmaceuticals from wastewaters: an overview. Carbohydr. Polym. Technol. Appl. 7, 100466. 10.1016/j.carpta.2024.100466

[B12] Da Silva Alves D. C. Healy B. Pinto L. A. D. A. Cadaval JR T. R. S. A. Breslin C. B. (2021). Recent developments in chitosan-based adsorbents for the removal of pollutants from aqueous environments. Molecules 26, 594. 10.3390/molecules26030594 33498661 PMC7866017

[B13] Fatullayeva S. Tagiyev D. Zeynalov N. Mammadova S. Aliyeva E. (2022). Recent advances of chitosan-based polymers in biomedical applications and environmental protection. J. Polym. Res. 29, 259. 10.1007/s10965-022-03121-3

[B14] Goci M. C. Leudjo Taka A. Martin L. Klink M. J. (2023). Chitosan-based polymer nanocomposites for environmental remediation of mercury pollution. Polymers 15, 482. 10.3390/polym15030482 36771779 PMC9921766

[B15] Goel S. (2017). Solid and hazardous waste management: an introduction. Adv. Solid Hazard. Waste Manag., 1–27. 10.1007/978-3-319-57076-1_1

[B16] González-Martínez A. De Simón-Martín M. López R. Táboas-Fernández R. Bernardo-Sánchez A. (2019). Remediation of potential toxic elements from wastes and soils: analysis and energy prospects. Sustainability 11, 3307. 10.3390/su11123307

[B17] Hajji S. Younes I. Ghorbel-Bellaaj O. Hajji R. Rinaudo M. Nasri M. (2014). Structural differences between chitin and chitosan extracted from three different marine sources. Int. J. Biol. Macromol. 65, 298–306. 10.1016/j.ijbiomac.2014.01.045 24468048

[B18] Ibitoye E. Lokman I. Hezmee M. Goh Y. Zuki A. Jimoh A. (2018). Extraction and physicochemical characterization of chitin and chitosan isolated from house cricket. Biomed. Mater. 13, 025009. 10.1088/1748-605x/aa9dde 29182521

[B19] Ibrahim N. A. Eid B. M. (2016). Potential applications of sustainable polymers in functionalization of cellulosic textile materials. Handb. Sustain Polym. Process Appl. 6, 215–264. 10.1002/9781119441632.ch44

[B20] Ibrahim N. A. Eid B. M. (2017). “Chitosan-based composite materials: fabrication and characterization,” in Handbook of composites from renewable materials. Editors Thakur V. K. Thakur M. K. Kessler M. R., 103–136.

[B21] Ibrahim N. Eid B. (2018). “Emerging technologies for source reduction and end-of-pipe treatments of the cotton-based textile industry,” in Handbook of textile effluent remediation. New York: Pan Stanford Publishing, Taylor and Francis Group, 185–202.

[B22] Kaczorowska M. A. Bożejewicz D. (2024). The application of chitosan-based adsorbents for the removal of hazardous pollutants from aqueous solutions—a review. Sustainability 16, 2615. 10.3390/su16072615

[B23] Kaya M. BağrıAÇıK N. Seyyar O. Baran T. (2015a). Comparison of chitin structures derived from three common wasp species (Vespa crabro Linnaeus, 1758, Vespa orientalis Linnaeus, 1771 and Vespula germanica (Fabricius, 1793)). Archives insect Biochem. physiology 89, 204–217. 10.1002/arch.21237 25850818

[B24] Kaya M. Baran T. Asan-Ozusaglam M. Cakmak Y. S. Tozak K. O. Mol A. (2015b). Extraction and characterization of chitin and chitosan with antimicrobial and antioxidant activities from cosmopolitan Orthoptera species (Insecta). Biotechnol. Bioprocess Eng. 20, 168–179. 10.1007/s12257-014-0391-z

[B25] Kaya M. Bitim B. Mujtaba M. Koyuncu T. (2015c). Surface morphology of chitin highly related with the isolated body part of butterfly (Argynnis pandora). Int. J. Biol. Macromol. 81, 443–449. 10.1016/j.ijbiomac.2015.08.021 26277749

[B26] Ke C.-L. Deng F.-S. Chuang C.-Y. Lin C.-H. (2021). Antimicrobial actions and applications of chitosan. Polymers 13, 904. 10.3390/polym13060904 33804268 PMC7998239

[B27] Kumari S. Annamareddy S. H. K. Abanti S. Rath P. K. (2017). Physicochemical properties and characterization of chitosan synthesized from fish scales, crab and shrimp shells. Int. J. Biol. Macromol. 104, 1697–1705. 10.1016/j.ijbiomac.2017.04.119 28472681

[B28] Li M. Li X. Wang L. Pei Y. An M. Liu J. (2021). Highly efficient and selective removal of anionic dyes from water using a cellulose nanofibril/chitosan sponge prepared by dehydrothermal treatment. J. Environ. Chem. Eng. 9, 105745. 10.1016/j.jece.2021.105745

[B29] Marei N. H. Abd El-Samie E. Salah T. Saad G. R. Elwahy A. H. (2016). Isolation and characterization of chitosan from different local insects in Egypt. Int. J. Biol. Macromol. 82, 871–877. 10.1016/j.ijbiomac.2015.10.024 26459168

[B30] Moder K. P. Russo J. P. Justiniano F. Marshall W. F. Mcghee T. H. Stankovich R. (2007). Development of a hazardous material compatibility storage guideline and tool. Process Saf. Prog. 26, 114–122. 10.1002/prs.10186

[B31] Mohan K. Ganesan A. R. Muralisankar T. Jayakumar R. Sathishkumar P. Uthayakumar V. (2020). Recent insights into the extraction, characterization, and bioactivities of chitin and chitosan from insects. Trends food Sci. Technol. 105, 17–42. 10.1016/j.tifs.2020.08.016 32901176 PMC7471941

[B32] Mondal P. Purkait M. K. (2018). Green synthesized iron nanoparticles supported on pH responsive polymeric membrane for nitrobenzene reduction and fluoride rejection study: optimization approach. J. Clean. Prod. 170, 1111–1123. 10.1016/j.jclepro.2017.09.222

[B33] Moria K. M. Khurshid H. Mustafa M. R. U. Alhothali A. Bamasag O. O. (2022). Application of the response surface methodology (RSM) in the optimization of acenaphthene (ACN) removal from wastewater by activated carbon. Sustainability 14, 8581. 10.3390/su14148581

[B34] Nayl A. A. Abd-Elhamid A. I. Arafa W. A. Ahmed I. M. El-Shanshory A. A. Abu-Saied M. A. (2022). Chitosan-functionalized-graphene oxide (GO@ CS) beads as an effective adsorbent to remove cationic dye from wastewater. Polymers 14, 4236. 10.3390/polym14194236 36236183 PMC9572660

[B35] Nouri M. Khodaiyan F. Razavi S. H. Mousavi M. A. (2016). The effect of different chemical and physical processing on the physicochemical and functional characterization of chitosan extracted from shrimp waste species of indian white shrimp. Prog. Rubber Plastics Recycl. Technol. 32, 39–54. 10.1177/147776061603200103

[B36] Olafadehan O. A. Amoo K. O. Ajayi T. O. Bello V. E. (2021a). Extraction and characterization of chitin and chitosan from Callinectes amnicola and Penaeus notialis shell wastes. J. Chem. Eng. Material Sci. 12, 1–30. 10.5897/jcems2020.0353

[B37] Olafadehan O. A. Amoo K. O. Ajayi T. O. Bello V. E. (2021b). Extraction and characterization of chitin and chitosan from Callinectes amnicola and Penaeus notialis shell wastes. J. Chem. Eng. Mater Sci. 12, 1–30. 10.5897/jcems2020.0353

[B38] Piekarska K. Sikora M. Owczarek M. Jóźwik-Pruska J. Wiśniewska-Wrona M. (2023). Chitin and chitosan as polymers of the future—obtaining, modification, life cycle assessment and main directions of application. Polymers 15, 793. 10.3390/polym15040793 36850077 PMC9959150

[B39] Qiao C. Ma X. Wang X. Liu L. (2021). Structure and properties of chitosan films: effect of the type of solvent acid. Lwt 135, 109984. 10.1016/j.lwt.2020.109984

[B40] Raut A. Satvekar R. Rohiwal S. Tiwari A. Gnanamani A. Pushpavanam S. (2016). *In vitro* biocompatibility and antimicrobial activity of chitin monomer obtain from hollow fiber membrane. Des. Monomers Polym. 19, 445–455. 10.1080/15685551.2016.1169379

[B41] Reshad R. A. I. Jishan T. A. Chowdhury N. N. (2021). Chitosan and its broad applications: a brief review. *Available at SSRN 3842055* .

[B42] Soares S. F. Amorim C. O. Amaral J. S. Trindade T. Daniel-Da-Silva A. L. (2021). On the efficient removal, regeneration and reuse of quaternary chitosan magnetite nanosorbents for glyphosate herbicide in water. J. Environ. Chem. Eng. 9, 105189. 10.1016/j.jece.2021.105189

[B43] Son Y.-J. Hwang I.-K. Nho C. W. Kim S. M. Kim S. H. (2021). Determination of carbohydrate composition in mealworm (*Tenebrio molitor* L.) larvae and characterization of mealworm chitin and chitosan. Foods 10, 640. 10.3390/foods10030640 33803569 PMC8002850

[B44] Tarique J. Sapuan S. Aqil N. Farhan A. Faiz J. Shahrizan S. (2023). A comprehensive review based on chitin and chitosan composites. Compos. Aquatic Environ., 15–66. 10.1007/978-981-19-5327-9_1

[B45] Teli M. Sheikh J. (2012). Extraction of chitosan from shrimp shells waste and application in antibacterial finishing of bamboo rayon. Int. J. Biol. Macromol. 50, 1195–1200. 10.1016/j.ijbiomac.2012.04.003 22522048

[B46] Thambiliyagodage C. Jayanetti M. Mendis A. Ekanayake G. Liyanaarachchi H. Vigneswaran S. (2023). Recent advances in chitosan-based applications—a review. Materials 16, 2073. 10.3390/ma16052073 36903188 PMC10004736

[B47] Torres F. G. Troncoso O. P. Pisani A. Gatto F. Bardi G. (2019). Natural polysaccharide nanomaterials: an overview of their immunological properties. Int. J. Mol. Sci. 20, 5092. 10.3390/ijms20205092 31615111 PMC6834193

[B48] Yan D. Li Y. Liu Y. Li N. Zhang X. Yan C. (2021). Antimicrobial properties of chitosan and chitosan derivatives in the treatment of enteric infections. Molecules 26, 7136. 10.3390/molecules26237136 34885715 PMC8659174

[B49] Yilmaz Atay H. (2019). Antibacterial activity of chitosan-based systems. Funct. chitosan drug Deliv. Biomed. Appl., 457–489. 10.1007/978-981-15-0263-7_15

[B50] Zainol Abidin N. A. Kormin F. Zainol Abidin N. A. Mohamed Anuar N. A. F. Abu Bakar M. F. (2020). The potential of insects as alternative sources of chitin: an overview on the chemical method of extraction from various sources. Int. J. Mol. Sci. 21, 4978. 10.3390/ijms21144978 32679639 PMC7404258

[B51] Zhu L. F. Chen X. Wu Z. Wang G. Ahmad Z. Chang M. W. (2020). Optimization conversion of chitosan from Ganoderma lucidum spore powder using ultrasound-assisted deacetylation: influence of processing parameters. J. Food Process. Preserv. 44, e14297. 10.1111/jfpp.14297

